# Simple and cost-effective UV spectrophotometric platforms integrating advanced green and blue metrics for concurrent analysis of dapagliflozin and vildagliptin in diabetes therapy

**DOI:** 10.1038/s41598-026-58523-w

**Published:** 2026-06-25

**Authors:** Eman Darweish, Hisham A. Hashem, Rawan Nasr, Eman A. Bahgat

**Affiliations:** 1 Pharmaceutical Chemistry Department, Faculty of Pharmacy, Egyptian Russian University, Badr City, 11829 Cairo Egypt; 2 Analytical Chemistry Department, Faculty of Pharmacy, Zagazig University, Zagazig, 44519 Egypt

**Keywords:** Dapagliflozin (DPF), Vildagliptin (VLT), Deconvoluted Fourier (DF), Click Analytical Chemistry Index (CACI), Chemistry, Health care

## Abstract

The co-administration of dapagliflozin (DPF) and vildagliptin (VLT) in tablet dosage forms is a clinically established strategy for managing of type 2 diabetes mellitus and has demonstrated favorable therapeutic outcomes. Nevertheless, the simultaneous quantitative determination of both drugs remains challenging because of the severe overlap in their UV absorption spectra, with λ_max_ values at 224 nm for DPF and 214 nm for VLT. In this study, novel eco-friendly UV spectrophotometric methods were developed and validated for the accurate determination of DPF and VLT in their combined pharmaceutical formulations. The proposed methods include Fourier deconvolution, mean centering, dual wavelength, and induced dual wavelength techniques, which allow precise, reliable, and sensitive analysis without the need for prior separation. These approaches are simple, minimize noise in ratio spectra, and require limited mathematical manipulation, thereby reducing analysis time. DPF and VLT showed linear responses over concentration ranges of 2–24 µg/mL and 10–100 µg/mL, respectively. The limits of detection ranged from 0.00486 to 0.7979 µg/mL for DPF and from 0.0185 to 0.8690 µg/mL for VLT. Method greenness was evaluated using Eco-scale, GAPI, MoGAPI, CaFRI, and blueness assessment, while CACI was applied to assess overall analytical performance. Statistical comparison between the methods showed no significant differences.

**Supplementary Information:**

The online version contains supplementary material available at 10.1038/s41598-026-58523-w.

## Introduction

 Diabetes may be a widespread health issue, but with continued research, education, and proactive management, we can confidently work towards preventing and controlling its impact on individuals and communities globally^1,2^. Type 2 diabetes mellitus (T2DM) is a condition that commonly manifests in adults as a result of a decline in insulin production and activity, causing elevated blood glucose levels. Despite the availability of various antidiabetic drugs to mitigate hyperglycemia, T2DM continues to be a major cause of mortality and morbidity. Individuals with this condition are at risk of developing severe health complications, such as foot ulceration and blindness, as well as cardiovascular and renal complications.

These complications demand diligent management and monitoring to prevent their progression and associated adverse outcomes^1^. During the management of T2DM, combination therapies have been proven to be a highly effective approach. They address several pathophysiological features of the condition and work synergistically to achieve a complementary impact on glucose-lowering. The utilization of these therapies has the potential to produce significant and transformative results for patients^3^.

In recent years, patients suffering from T2DM have increasingly utilized dipeptidyl peptidase-4 (DPP-4) inhibitors, such as VLT to manage their blood sugar levels and prevent the breakdown of glucagon-like peptide-1 (GLP-1). It is important to note, however, that insulin-dependent antidiabetic medication alone may not effectively lower glycated hemoglobin (HbA1c) levels^4^. As a result, physicians often prescribe a complementary treatment approach that includes recently developed sodium glucose cotransporter-2 (SGLT-2) inhibitors as DPF. These medications regulate glucose reuptake in the kidneys, increasing renal glucose excretion by inhibiting it^5^.

**Fig. 1:**
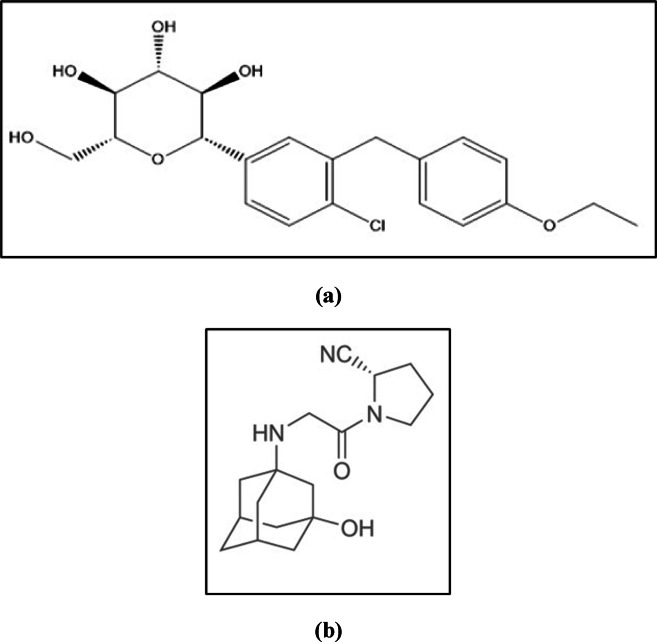
Chemical structure of DPF **(a)** & VLT **(b)**.

The fixed-dose combination of DPF, (2 S,3R,4R,5 S,6R)−2-[4-chloro-3-[(4- ethoxyphenyl)methyl]phenyl]−6-(hydroxymethyl)oxane-3,4,5-triol; (2 S)-propane-1,2-diol; hydrate and VLT, (S)−1-[N-(3-hydroxy-1-adamantyl) glycyl] pyrrolidine-2-carbonitrile (Fig. 1**)**^6,7^, showed an excellent cardioprotective impact along with lowered HbA1c levels when compared with single regimens^8^. For patients diagnosed with type 2 diabetes, the combination of SGLT2 inhibitors, such as DPF, and DPP4 inhibitors, such as VLT, has shown potential for reducing the incidence of genitourinary infections (GTIs)^9^.

Both drugs have been reported to have kidney-beneficial effects by lowering urinary albumin excretion, which is a significant risk factor for renal diseases. Notably, these drugs are known to produce significantly fewer side effects than other oral hypoglycemic agents such as, hypoglycemia and weight gain^10^.

Various analytical approaches have been utilized for the measurement of DPF and VLT in their pharmaceutical formulations and biological samples, either alone or in combination with other elements. According to literature, DPF was determined using UV spectroscopy^11–13^, spectrofluorimetry^14^, capillary electrophoresis^15^, LC-MS/MS^16^ and HPLC^2,17–19^. VLT was also assessed by UV spectroscopy^20,21^, capillary electrophoresis^22^, spectrofluorimetry^23^, LC-MS/MS and HPLC^24,25^. Few techniques have been utilized to determine the combination of DPF and VLT in a pharmacological dosage form such as spectroscopic^1,26^ and HPLC^27^ methods.

Therefore, this study aimed to develop and validate simple, cost-effective, eco-friendly, and sensitive UV spectrophotometric methods for the simultaneous determination of DPF and VLT in their combined pharmaceutical dosage forms.

Green analytical chemistry aims to minimize the environmental impact of analytical procedures through reducing hazardous chemicals, waste generation, and energy consumption^28^. Several greenness tools, including Eco-scale^29^, GAPI^30^, MoGAPI^31^ and CaFRI^32^ tools were used to assess the greenness of the suggested analytical methodologies in addition to the blueness assessment^33^.

Click Analytical Chemistry Index (CACI) is a practical and user-friendly tool used for the qualitative and quantitative evaluation of analytical methods based on simplicity, rapidity, reliability, and real-world applicability^34^.

Accordingly, the proposed Fourier deconvolution, mean centering, dual wavelength, and induced dual wavelength spectrophotometric methods were successfully developed and evaluated using advanced green, blue, and practical analytical assessment metrics.

## Experimental

### Materials and reagents

DPF and VLT pure standards were gratefully acquired from Marcyrl for pharmaceutical industries and Mash premier for pharmaceutical industries (Cairo, Egypt) respectively. Purity analyses revealed values of 99.80% for DPF and 99.79% for VLT. Ethanol was obtained from Sigma-Aldrich (USA) and its purity was assessed to be 99.80%. **Dapagold V10**® tablets (B.N.MT22A424D) claimed to contain 10 mg DPF and 100 mg VLT.

### Instruments and software

A dual-beam UV-Vis spectrophotometer (model V-630, Jasco, Japan) controlled by Spectra Manager II software was used to perform the spectrophotometric analyses. The instrument was operated at a scanning speed of 1000 nm/min with a spectral slit width of 2 nm. Mean centering (MC) was developed and calculated utilizing MATLABVR R2013b (8.2.0.701).

### Preparation of stock standard and working solutions

DPF and VLT standard solutions, each with 1 mg/mL concentration, were cautiously produced by dissolving 100 mg of DPF and VLT in individual 100 mL volumetric flasks, each having 50 mL of ethanol. Each flask was subsequently subjected to careful agitation and sonication for a duration of 10 min. Upon allowing the flask to settle, the volume was subsequently adjusted to the designated mark with ethanol. Working solutions at a concentration of 100 mg/mL for both standards were carefully prepared by transferring 10 mL from each stock solution into a 100 mL volumetric flask, then adding ethanol to reach the final volume. Each of the stock standard and working solutions was maintained at a temperature of 4 degrees Celsius until use.

### Calibration curves construction

Ethanol was used to prepare standard serial solutions of DPF and VLT at concentrations ranging from 2 to 22 µg/mL and 10 to 120 µg/mL, respectively. After that, all solutions were scanned between 200 and 400 nm, and ethanol was used as a blank to record the zero-order absorption spectra.

#### Determination of DPF and VLT using deconvoluted Fourier method (DF)

The spectra of DPF and VLT standards were deconvoluted using the Fourier deconvolution function of the spectrophotometer software, applying the full width at half maximum value. The DPF amplitude was recorded at 207 nm, where VLT showed a zero crossing at the specified wavelength. In contrast, the amplitude of VLT was recorded at 218 nm, where DPF exhibited a zero crossing. The amplitudes of deconvoluted spectra for DPF and VLT at the specified wavelength were graphed against their respective concentrations to generate calibration curves. Subsequent calculations were performed using the regression equations.

#### Determination of DPF and VLT using mean centering method (MC)

DPF zero-order spectra were recorded and divided by VLT (50 µg/mL) as the divisor, while VLT zero-order spectra were divided by DPF (15 µg/mL) as the divisor. The stored ratio spectra within the range of 200–400 nm were mean-centered using MATLAB VR R2013b (8.2.0.701) software. Subsequently, calibration curves were created by plotting the amplitudes at 234 nm for DPF and at 292 nm for VLT against their respective concentrations.

#### Determination of VLT using dual-wavelength method (DW)

In presence of DPF, dual wavelength method was used to determine VLT. After having zero order absorption spectra of VLT concentrations recorded, the amplitudes of absorbance difference were measured at 221 and 228 nm for VLT, where the absorbance difference of DPF was zero, then plotted against their respective concentrations in (µg/mL). After that, VLT concentrations were calculated using the regression equation.

#### Determination of DPF using induced dual-wavelength method (IDW)

Each concentration of DPF had its zero-order absorption spectrum recorded. An equality factor (F_eq_) for VLT was calculated by dividing the absorbance at 230 nm by the absorbance at 236 nm, and all absorbance values for DPF at 236 nm were multiplied by the F_eq_ (236 nm*Feq). The amplitude difference of DPF at 230 nm and 236 nm*F_eq_ was plotted against their respective concentrations in (µg/mL).

### Analysis of laboratory prepared mixtures

Different binary mixtures with varying ratios of DPF and VLT were laboratory produced and examined. After preparing the various mixture ratios, the spectra of the mixtures were measured and analyzed according to the methods described previously (Table 1).

**Table 1:** Analysis of DPF and VLT in laboratory prepared mixtures.

Concentration (µg/mL)		%Recovery					
		DF		MC		DW	IDW
DPF	VLT	DPF	VLT	DPF	VLT	VLT	DPF
3	15	102.66	99.06	98.75	98.04	100.76	99.22
5	50	101.32	99.66	101.23	102.77	98.70	100.61
1	20	99.79	102.18	100.39	102.89	101.54	98.23
2	60	98.30	101.12	99.20	98.89	98.57	101.33
4	20	99.83	101.74	102.63	101.94	100.03	98.91

### Assay of the pharmaceutical dosage form

The fixed-dose combination tablets of VLT and DPF were precisely weighed and ground into a powder. A sufficient quantity of ground powder was then added to a 100-mL volumetric flask having 50 mL of ethanol. The solution was dissolved, sonicated for 10 min, and then filtered into a volumetric flask. The remaining tablet powder was filtered with fresh ethanol, and additional fresh ethanol was added to achieve a total volume of 100 mL. Additional dilutions were applied to achieve the necessary concentrations within the calibration curves for the specified drugs. Absorption signal was recorded in the UV wavelength range (200–400 nm) and analyzed using the methods previously outlined. Finally, the analytes were quantified through the application of the respective linear equations. The standard addition method was employed, and the percentage recovery of the added analytes was determined (Table 2).

**Table 2:** Application of standard addition technique for the analysis of **Dapagold V10**® tablets.

Drug	Concentration(µg/mL)	Pure added (µg/mL)	found ^a^ concentration (µg/mL)				Recovery ^b^ (*R*%)			
			DF	MC	IDW	DW	DF	MC	IDW	DW
DPF	10	4	9.94	10.02	10.12	-	100.73	100.03	102.42	-
		8					98.38	99.73	99.61	-
		12					100.36	98.841	100.72	-
		Mean ±					99.82 ± 1.26	99.53 ± 0.61	100.92 ± 1.39	-
		RSD%								
VLT	100	10	100.11	101.79	-		99.86	100.48	-	100.82
		15					98.95	99.92	-	99.98
		20					101.40	100.69	-	100.73
		Mean ±					100.14 ± 1.22	100.36 ± 0.39	-	100.51 ± 0.45
		RSD%								

^a^ Mean of three determinations.

^b^ Mean of three determinations.

## Results and discussion

UV spectrophotometric techniques allow for accurate and straightforward differentiation between drugs with significantly overlapping spectra in a short time period, particularly in quality control laboratories where other expensive instruments may not be readily available. There have been no sufficient reported spectrophotometric techniques to resolve the strong overlap between signals of both DPF and VLT. Compared with conventional chromatographic methods, the proposed spectrophotometric methods offer reduced analysis time owing to the absence of chromatographic separation, mobile phase preparation, and column equilibration steps. Additionally, the proposed methods provide a more sustainable analytical approach through reduced solvent consumption and minimized waste generation^35^.

From an analytical perspective, the suggested methods can be considered indirect optical sensing systems, where the UV absorption signal of each drug is used as the analytical response. Instead of relying on physical separation, these approaches depend on mathematical manipulation of the recorded spectra to resolve the severe overlap between DPF and VLT. This signal processing enables the extraction and resolution of each analyte’s contribution from the same spectral data, allowing their simultaneous quantification within the same sample.

Consequently, the three developed methods introduce a chance for determining the concentration of the chosen drug and resolve this severe overlap in their dosage form using green, blue, accurate, reliable and simple spectrophotometric methods.

### Determination of DPF and VLT using deconvoluted fourier method (DF)

DF is regarded a simple, cost-effective, and reliable spectrophotometric technique for determining strongly overlapping spectra without the necessity for prior separation. This method involves computation to deal with the challenge of strongly overlapping spectral signals from DPF and VLT by compressing the bandwidth, enabling their differentiation^36^. This technique relies on minimal mathematical manipulation of the zero-order spectra of DPF and VLT, allowing for straightforward evaluation of DPF in presence of VLT and vice versa.

**Fig. 2:**
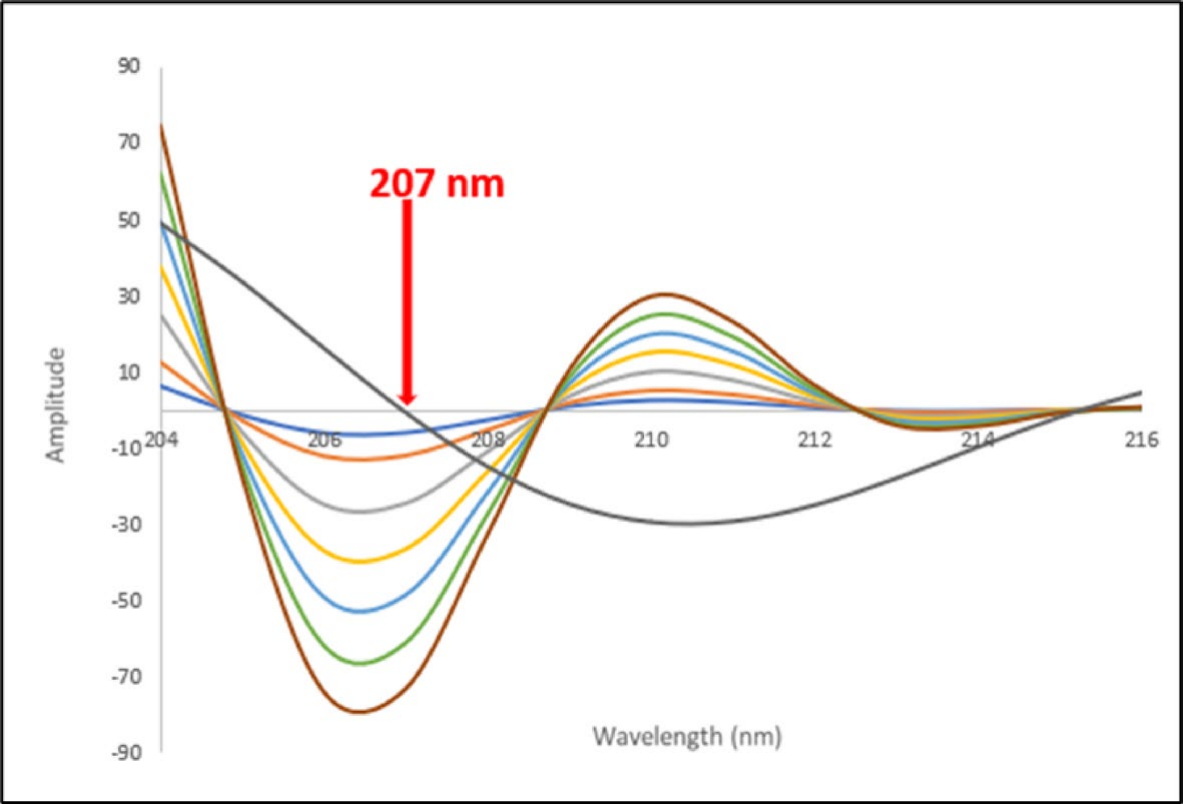
Deconvoluted spectra of DPF (2–24 µg/mL) calculated at 207 nm zero-crossing point of VLT deconvoluted spectrum.

**Fig. 3:**
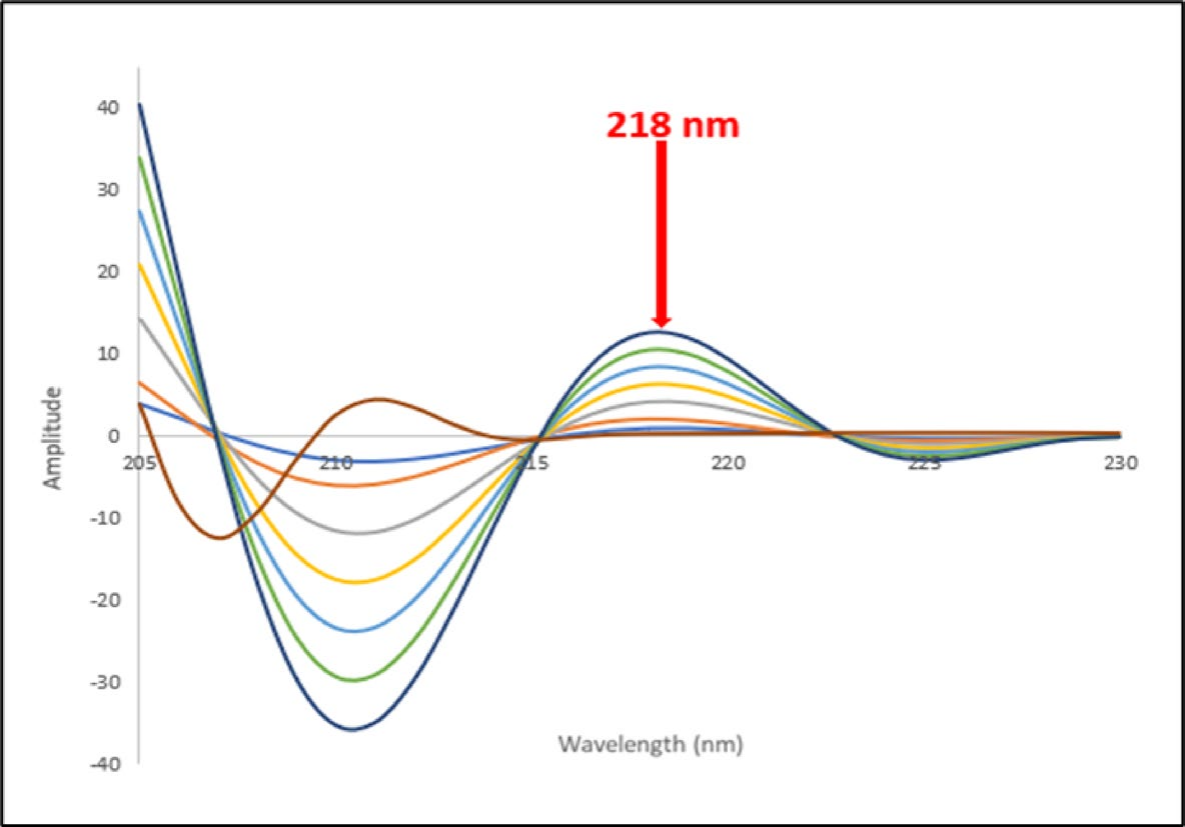
Deconvoluted spectra of VLT (10–120 µg/mL) calculated at 218 nm zero-crossing point of DPF deconvoluted spectrum.

The deconvolution process is carried out utilizing the Fourier self-deconvolution function in the spectrophotometer software, with a full width at half maximum (FWHM) of 18^37^. Following the application of the Fourier method, the DPF amplitude was recorded at 207 nm as shown in Fig. 2, indicating no crossing with the VLT, while the VLT amplitude was noted at 218 nm in Fig. 3, also showing no crossing with the DPF. Calibration curves were generated by plotting the deconvoluted spectra amplitudes of DPF and VLT at 207 and 218 nm against their respective concentrations. Table 3 presents the computed regression equations.

### Determination of DPF and VLT using mean centering method

**Fig. 4:**
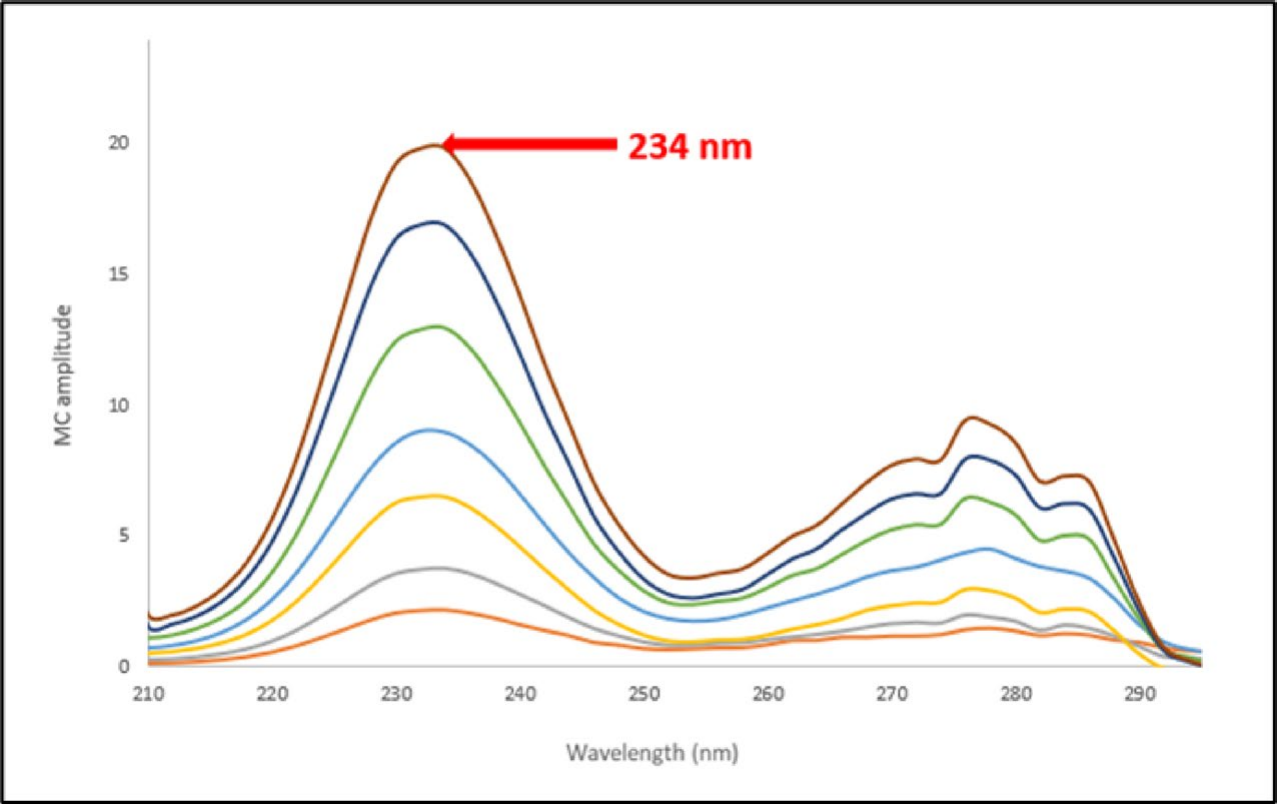
Mean-centered ratio spectra of DPF (2–24 µg/mL) using the spectrum of 50 µg/mL VLT as a divisor.

**Fig. 5:**
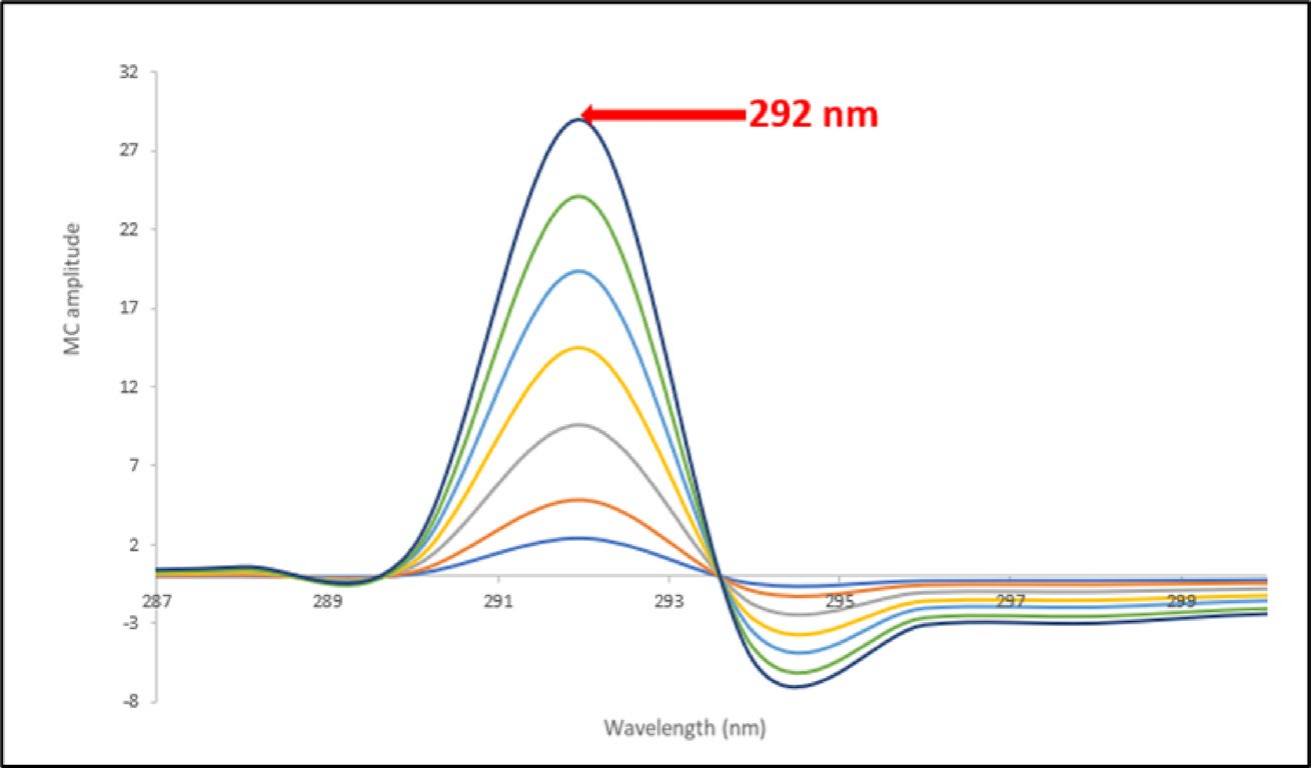
Mean-centered ratio spectra of VLT (10–120 µg/mL) using the spectrum of 15 µg/mL DPF as a divisor.

A novel and straightforward method for the co-current determination of binary mixtures was developed, bypassing the necessity for preliminary separation processes. MC of ratio spectra was applied for resolving the spectral overlap between DPF and VLT. Unlike derivative spectrophotometric methods that may amplify spectral noise during derivative transformation, the proposed mean-centering approach minimizes noise interference by avoiding derivative processing steps^38^. The stored ratio spectra in 200–400 nm range were mean-centered. Then the peak amplitude at 234 nm for DPF (Fig. 4**)** and 292 nm for VLT (Fig. 5**)** were used to calculate the concentrations after using their regression equations.

The noise-reducing capability of the proposed MC method was further demonstrated by comparison with a previously reported first-derivative spectrophotometric method for the simultaneous determination of DPF and VLT^39^. The reported method exhibited intra-day %RSD values of 1.58 and 0.55 for DPF and VLT, respectively, whereas the proposed MC method achieved lower values of 1.21 and 0.334 **(**Table 3**)**, indicating improved signal stability and reduced noise interference.

### Determination of VLT using dual-wavelength method (DW)

**Fig. 6:**
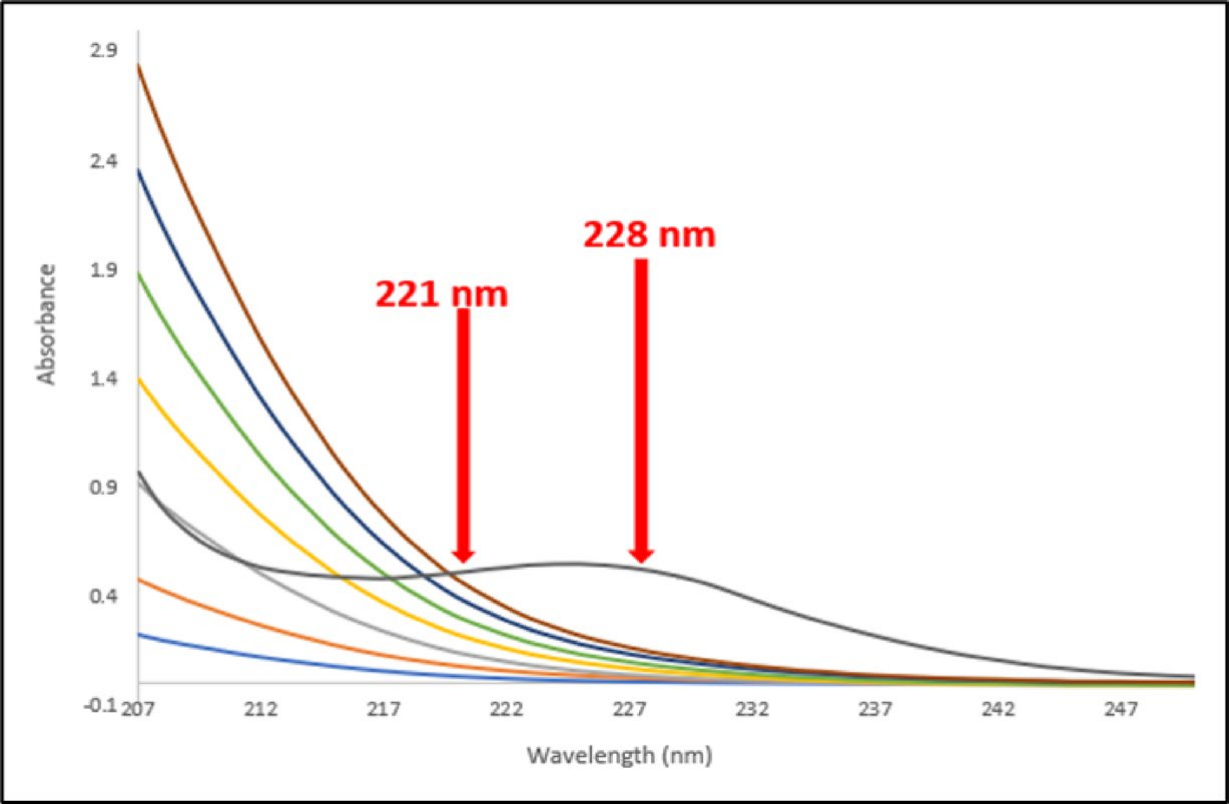
Zero order absorption spectra for dual wavelength method of 10–120 µg/mL VLT in presence of DPF.

The dual-wavelength approach focuses on the theory that the difference of absorbance between two wavelengths in a mixture spectrum of the drug of interest is directly proportional to its concentration, where there is no interference from the other drug. This method was applied to determine VLT in zero-order spectra by obtaining the difference in absorbance between 221 nm and 228 nm (∆A 221–228), where the best sensitivity to assess VLT was shown as DPF showed no absorbance (Fig. 6). The obtained results are recorded in Table 3 after using their regression equations.

### Determination of DPF using induced dual-wavelength method (IDW)

**Fig. 7:**
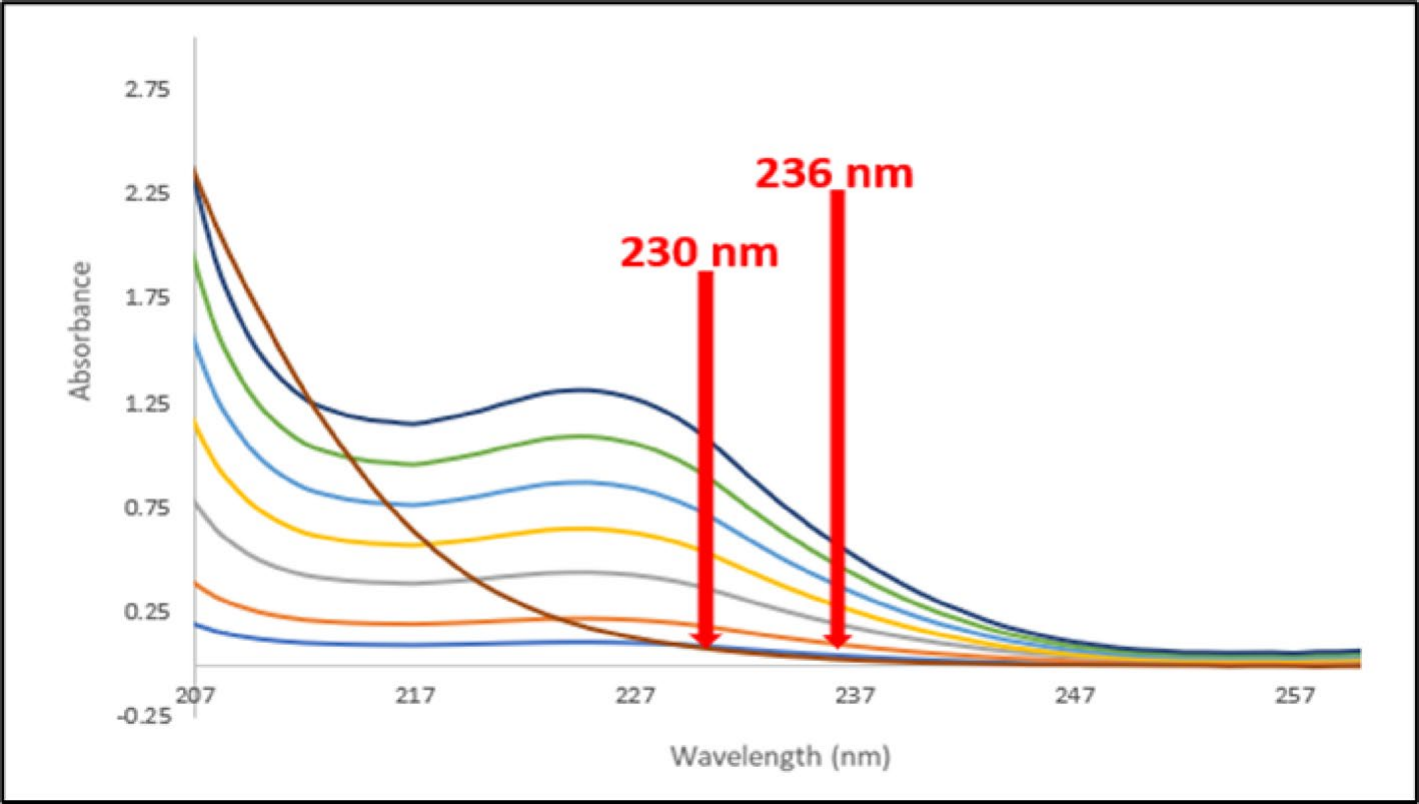
Zero order absorption spectra for induced dual wavelength of 2–24 µg/mL DPF in presence of VLT where Feq for VLT = abs (230)/abs (236).

The IDW technique was utilized in situations where the conventional DW approach could not be applied. The DW method involves selecting two specific wavelengths where the subtraction of one drug (X) yields a difference of zero. This enabled the determination of drug Y without any interference from drug X at these wavelengths. However, this was not applicable to DPF in its combination with VLT, as the difference for VLT was not zero at the two precisely chosen wavelength points of 230 and 236 nm. Consequently, its interfering effect persisted (Fig. 7**)**. Therefore, IDW determined a solution by calculating an equality factor (F_eq_) for VLT.

The average value of F_eq_ for VLT was calculated by dividing the absorbance at 230 nm by the absorbance at 236 nm. To prevent the confounding influence of VLT in the mixture, the amplitude values for DPF at 230 and 236 nm were recorded by subsequently multiplying all absorbance values for DPF at 236 nm by the corresponding F_eq_^35^. A calibration curve was created by plotting DPF concentrations against absorbance difference (∆A 230.0–236.0 nm * F_eq_).

A regression equation was used to calculate pure DPF concentration. The calculated equality factor showed consistent reproducibility throughout the studied concentration ranges, as evidenced by the low %RSD values (< 2%) obtained during validation studies (Table 3**)**.

Overall, all the proposed methods enabled the simultaneous determination of both drugs from the same spectral measurement without prior separation, confirming their suitability as simple and effective analytical sensing approaches for multicomponent pharmaceutical analysis.

## Method validation

Validation parameters of the reported approaches were all validated in accordance with the ICH Q2 (R1) recommendations^40^.

### Linearity and range

Linearity for DPF and VLT in the proposed spectrophotometric techniques was assessed by determining concentrations within the ranges of 2–24 µg/mL for DPF and 10–120 µg/mL for VLT, as detailed in Table 3. These selected concentration ranges were chosen to match the expected concentrations during pharmaceutical dosage form analysis. The determination of each concentration was carried out three times, and the resulting regression equations were presented in Table 3. The methods employed demonstrated strong linearity, indicated by a high coefficient of determination and a low %RSD.

### Limit of detection (LOD) and Limit of quantification (LOQ)

LOD and LOQ were calculated by identifying the lowest concentrations that could be detected and quantitatively measured according to the recommendations of ICH Q2 (R1) using the following equations:

$${\rm{LOD }} = {\rm{ 3}}.{\rm{3 }}\sigma /{\rm{S}}$$

$${\rm{LOQ }} = {\rm{ 1}}0{\rm{ }}\sigma /{\rm{S}}$$

where σ represents the standard deviation of the intercept and S is the slope of the calibration curve. The low obtained LOD and LOQ values indicate the high sensitivity of the developed spectrophotometric methods. Sensitivity was further evaluated based on the slope values of the corresponding calibration equations. The obtained results are presented in Table 3.

### Accuracy

The developed methods were evaluated for accuracy by analyzing three different concentrations of DPF and VLT, followed by calculating the mean percentage recovery, as shown in Table 3.

### Precision

To ensure the precision of the methods used for the simultaneous quantification of drugs in their binary formulations, three different concentrations were analyzed in triplicate both within a single day and across three consecutive days. This was employed to assess repeatability and intermediate precision. The resulting precision values, expressed as %RSD, were summarized in Table 3, demonstrating that the proposed methods for the analysis of DPF and VLT were precise.

**Table 3:** Validation data for analysis of DPF and VLT.

Validation parameter	DF		MC		IDW	DW
	DPF	VLT	DPF	VLT	DPF	VLT
Wavelength (nm)	207	218	234	292	230 and 236	221 and 228
Linearity range (µg/mL)	2–24	10–120	2–24	10–120	2–24	10–120
Slope	3.0481	0.1061	0.8118	0.241	0.0246	0.0022
Intercept	−0.002	−0.047	0.306	−0.001	−0.0001	−0.0009
Correlation Coefficient	0.999	0.999	0.9993	0.999	0.999	0.999
Accuracy (recovery% ± SD) ͣ	99.81 ± 0.218	100.76 ± 1.052	99.85 ± 0.981	99.93 ± 0.762	99.97 ± 0.699	100.36 ± 0.725
Precision						
Intra- day ^b^ (% RSD)	0.471	0.299	1.21	0.334	0.086	1.74
Inter- day ^c^ (% RSD)	1.95	0.561	0.922	0.503	0.466	0.420
LOD (µg/mL)	0.0048	0.869	0.797	0.0185	0.0198	0.863
LOQ (µg/mL)	0.0144	2.607	2.391	0.0555	0.0594	2.589

^a^ Mean of five determinations.

^b^ Mean of three various concentrations for DPF and VLT recurred repeated three times within the same day.

^c^ Mean of three various concentrations for DPF and VLT recurred repeated three times within three successive days.

## Statistical analysis

The suggested methods for analyzing DPF and VLT in pharmaceutical preparation were effectively practiced and compared to the recorded HPLC method using F-tests and t-tests, respectively^41^. As shown in Table 4, the comparison between the proposed and reported methods revealed no significant statistical difference. Statistical comparison between the proposed and reported methods was performed using five determinations (*n* = 5). To make the precision and variability of our results easier to see, we created error-bar plots (mean ± SD) from the statistical data in Table 4. The charts (Figure S2) clearly show how closely the proposed methods align with the reported ones, while also highlighting that some of our spectrophotometric approaches actually offer noticeably lower variability.

**Table 4:** Statistical analysis of the adopted spectrophotometric methods and the reported method for simultaneous determination of DPF and VLT mixtures.

Parameters	DPF				VLT			
	Reported^41^ ^a^	DF	MC	IDW	Reported^41^ ^a^	DF	MC	DW
Mean	99.96	99.86	99.58	100.50	98.93	100.05	100.49	100.25
SD	1.21	0.91	0.45	1.19	0.99	0.96	0.40	0.53
Variance	1.46	0.82	0.20	1.41	0.98	0.92	0.16	0.28
t-test	-	0.15	0.66	0.71	-	1.82	3.27	2.63
f-test	-	1.77	7.23	1.03	-	1.06	6.13	3.49

The theoretical values of t and f at *p* = 0.05 are (2.31) and (6.38), respectively where *n* = 5.

^a^ The reported RP-HPLC method for simultaneous estimation of DPF and VLT.

## Greenness and blueness evaluation

To understand the impact of established analytical methods on animal and environmental health, their greenness and blueness must be evaluated. Several quantitative and semi-quantitative greenness and blueness assessment techniques were available to assess environmental sustainability. Greenness and blueness were evaluated in this study using the eco-scale, GAPI, MoGAPI and CaFRI and BAGI methods.

### Assessment of greenness of the analytical methods using analytical eco-scale

The greenness of the developed methods was evaluated using the Analytical Eco-Scale, a semi-quantitative tool based on penalty points assigned to reagents, instrumentation, energy consumption, and waste generation^42,43^. The obtained Eco-Scale score was 90, indicating excellent environmental compatibility. Minor penalty points were mainly attributed to ethanol usage and waste treatment, as presented in Table 5.

**Table 5:** Assessment of the greenness of the analysis using the eco-scale tool.

Reagents	Signal of wards	Number of pictograms	Penalty points
Ethanol	2	2	4
Instrument	Penalty points		
Energy (UV/VIS spectrophotometer) (< 0.1 kWh per sample)	0		
Occupational hazards Analytical process hermetization	0		
Waste 1–10 mL, no treatment	6		
Total Penalty points	Ʃ10		
Analytical eco-scale total score ^a, b^ Excellent green analysis	90		

^a^ Analytical Eco-scale total score = 100-total penalty points.

^b^ If the score is > 75, it indicates excellent green analysis. If the score is > 50, it indicates acceptable green analysis.

If the score is < 50, it indicates inadequate green analysis.

### Assessment of greenness of the analytical methods using GAPI

GAPI was employed to evaluate the environmental impact of our developed analytical procedures throughout the entire workflow, including sample preparation, reagent usage, instrumentation, energy consumption, and waste generation^30,44^. The resulting GAPI pictogram (Figure S3) showed predominantly green and yellow zones, indicating low to moderate environmental impact. The yellow zones were mainly associated with solvent usage, storage conditions, and moderate energy consumption, whereas the only red zone was attributed to the absence of waste treatment.

### Assessment of greenness of the analytical methods using MoGAPI

MoGAPI was applied as a hybrid greenness assessment tool combining the visual evaluation of GAPI with the quantitative scoring system of the Analytical Eco-Scale^45^. The obtained MoGAPI pictogram (Figure S4) and score 87 confirmed the excellent environmental sustainability of the suggested UV spectrophotometric methods.

### Assessment of greenness of the analytical methods using CaFRI

CaFRI was employed to evaluate the carbon footprint and environmental sustainability of our analytical methods based on parameters related to energy consumption, waste generation, transportation, and chemical usage^32,46,47^. The obtained CaFRI pictogram and cumulative score of 88 demonstrated the favorable environmental performance and reduced carbon footprint of the proposed spectrophotometric methods as in Figure S5.

### Assessment of blueness of the analytical methods

The practicality and applicability of analytical methods were evaluated using BAGI^33,48^. With a remarkable BAGI score of 77.5 Figure S6, our UV approaches have demonstrated exceptional practicality and applicability. This unequivocal evidence proves that the proposed techniques are not only effective but also essential for achieving optimal results.

## Assessment of the analytical methods using CACI

**Fig. 8:**
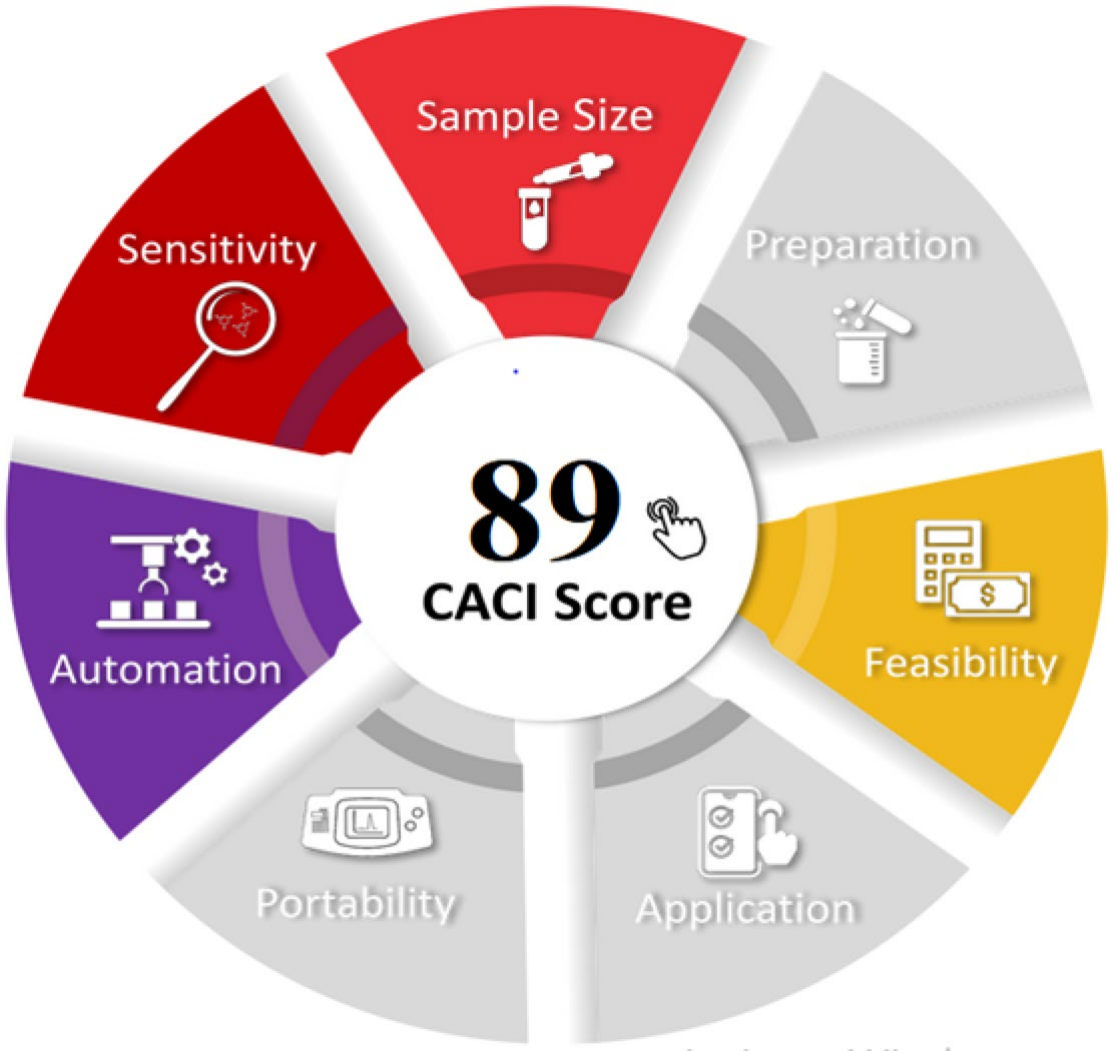
Assessment profile for the evaluated analytical procedures utilizing the CACI tool.

CACI assessment was employed to evaluate the operational practicality and analytical applicability of the proposed methods based on several performance-related criteria^34^. Before CACI can be evaluated, it is important to make sure that the technique is valid. Performance in each category is visually represented through a color-coded pictogram: a colored segment indicates high performance, gray reflects moderate or acceptable performance, and black denotes inadequate performance or non-compliance with the specified criteria. The developed UV methods have shown highest use-friendliness and practicality with CACI score of 89 as shown in Fig. 8.

## Conclusions

Innovative techniques have been developed to simultaneously determine DPF and VLT, using novel DF, DW, and MC approaches. These techniques have never been used before for DPF or VLT determination. The procedures have been optimized and validated in compliance with ICH standards, ensuring their reliability and accuracy. The benefits of these new techniques include their simplicity, requiring only minimal mathematical adjustments to the zero-order spectra. Additionally, the new spectrophotometric techniques are more environmentally friendly than the previous HPLC method due to their improved “greenness” and blueness features. Applying the novel CACI tool also ensures practicality and user-friendliness of our methods. UV spectroscopy provides a lower-cost alternative to liquid chromatography for regular quality assessment of a binary formulation containing DPF and VLT, making it an ideal choice for pharmaceutical companies that want to ensure quality while reducing costs.

## Supplementary Information

Below is the link to the electronic supplementary material.

Supplementary Material 1

## Data Availability

Data available on request All data supporting the findings of this study are available within the paper and its supplementary information. For further information regarding the data used in this study, please contact: [Rawan Nasr Mohamed], [[rawan-nasr@eru.edu.eg] (mailto: rawan-nasr@eru.edu.eg)].
