# Supplementary material for: Simple and cost-effective UV spectrophotometric platforms integrating advanced green and blue metrics for concurrent analysis of dapagliflozin and vildagliptin in diabetes therapy
Source: Sci Rep. 2026 Jun 25;16:19604. doi: 10.1038/s41598-026-58523-w (PMC13303919; doi:10.1038/s41598-026-58523-w)
Supplement: Supplementary file 1 — Supplementary Material 1 [file 41598_2026_58523_MOESM1_ESM.docx]

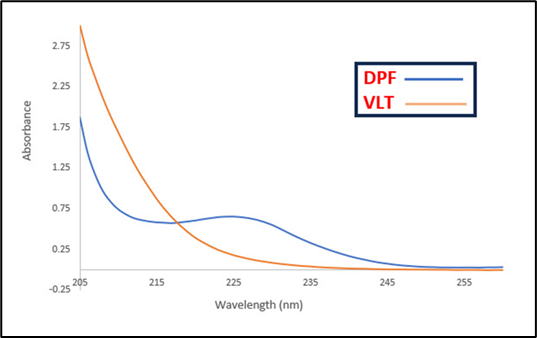


**Figure S1: Zero absorption spectra of 12 µg/mL DPF and 100 µg/mL VLT in ethanol.**

**
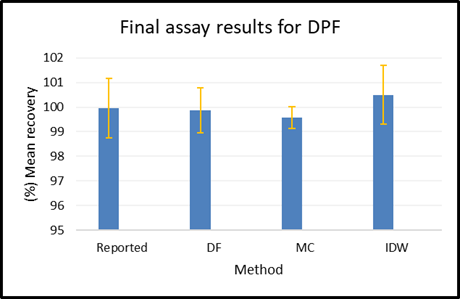
**

**(a)**

**
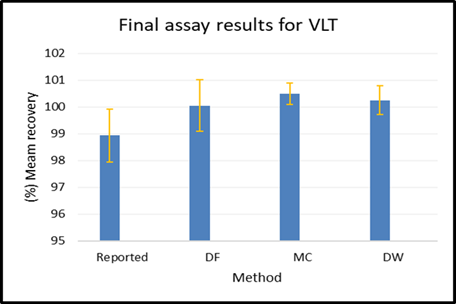
**

**(b)**

**Figure S2: Error bar charts comparing the final assay results obtained for DPF (a) and VLT (b) using the suggested and reported methods.**

**
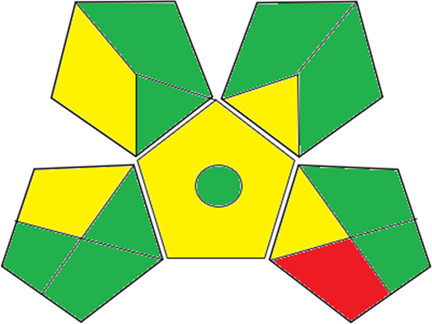
**

**Figure S3: Assessment profile for the evaluated analytical procedures utilizing the GAPI tool.**

**
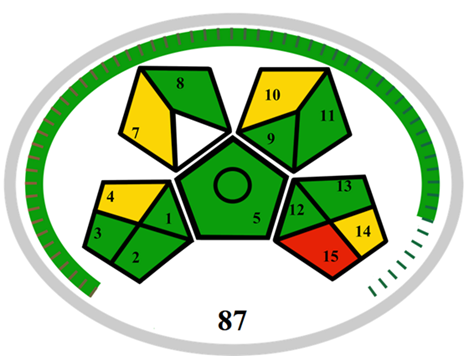
**

**Figure S4: Assessment profile for the evaluated analytical procedures utilizing the MoGAPI tool.**

**
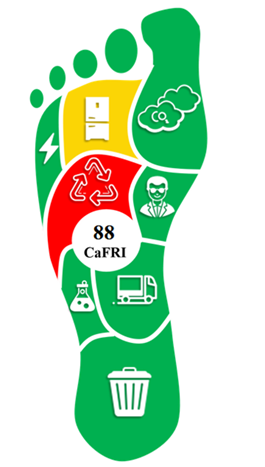
**

**Figure S5: Assessment profile for the evaluated analytical procedures utilizing the CaRFI tool.**

**
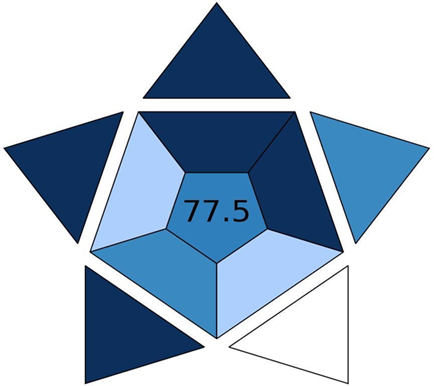
**

**Figure S6: Assessment profile for the evaluated analytical procedures utilizing the BAGI measure.**
